# RETRACTED: Kim et al. Inhibition of EphA2 by Dasatinib Suppresses Radiation-Induced Intestinal Injury. *Int. J. Mol. Sci.* 2020, *21*, 9096

**DOI:** 10.3390/ijms27114653

**Published:** 2026-05-22

**Authors:** Areumnuri Kim, Ki Moon Seong, You Yeon Choi, Sehwan Shim, Sunhoo Park, Seung Sook Lee

**Affiliations:** 1 Laboratory of Radiation Exposure and Therapeutics, National Radiation Emergency Medical Center, KIRAMS, Seoul 01812, Republic of Korea; ssh3002@kirams.re.kr (S.S.); sunhoo@kirams.re.kr (S.P.); sslee@kirams.re.kr (S.S.L.); 2 Laboratory of Biodosimetry, National Radiation Emergency Medical Center, KIRAMS, Seoul 01812, Republic of Korea; skmhanul@kirams.re.kr (K.M.S.); c9640@kirmas.re.kr (Y.Y.C.)

The journal retracts the article, “Inhibition of EphA2 by Dasatinib Suppresses Radiation-Induced Intestinal Injury” [1], cited above.

Following publication, concerns were brought to the attention of the Editorial Office regarding the integrity of the figures presented in this publication [1].

In accordance with the journal’s standard procedures, the Editorial Office and Editorial Board conducted an investigation that identified indications of inappropriate editing and duplication within images presented in Figures 3, 5A and 6, including a duplication with images from an earlier publication [2], presenting different experimental conditions. Despite repeated attempts to contact the authors, no response was received. Consequently, the Editorial Board has lost confidence in the reliability of the reported findings and has decided to retract this publication, as per MDPI’s retraction policy.

This retraction was approved by the Editor-in-Chief of the journal *IJMS*.

The authors have been informed of this retraction but did not provide a comment on this decision.
